# Inflammasomes: novel therapeutic targets for metabolic syndrome?

**DOI:** 10.3389/fendo.2025.1569579

**Published:** 2025-05-13

**Authors:** Pengyu Cao, Yulin Yang, Ningning Zhang, Bojian Wang, Zhenwei Gong

**Affiliations:** ^1^ The Second People’s Hospital of Changzhou, the Third Affiliated Hospital of Nanjing Medical University, Changzhou, Jiangsu, China; ^2^ Changzhou Medical Center, Nanjing Medical University, Changzhou, Jiangsu, China; ^3^ School of Nursing, Jilin University, Changchun, Jilin, China; ^4^ Division of Endocrinology, Department of Pediatrics, Children’s Hospital of Pittsburgh of UPMC, University of Pittsburgh School of Medicine, Pittsburgh, PA, United States

**Keywords:** obesity, diabetes, dyslipidemia, inflammation, innate immunity, interleukins

## Abstract

Chronic inflammation is a hallmark for Metabolic Syndrome (MetS). It is also one of the most important risk factors for insulin resistance and metabolic disorders. Inflammasomes, which are intracellular multiprotein complexes within the innate immune system, regulate the production and maturation of pro-inflammatory cytokines including interleukin-1β (IL-1β) and IL-18 upon sensing pathogens or danger signals in the cytosol. A growing body of evidence indicates that inflammasomes play a pivotal role in the pathophysiology and progression of metabolic diseases, as deficiency in the key component of inflammasomes protects mice from high fat diet induced obesity and insulin resistance. Thus, in this review, we will summarize the role of inflammasomes in MetS and how to treat MetS by targeting inflammasomes. This may provide novel insights and therapeutic targets for treating metabolic disorders.

## 1 Introduction

**Figure 1:**
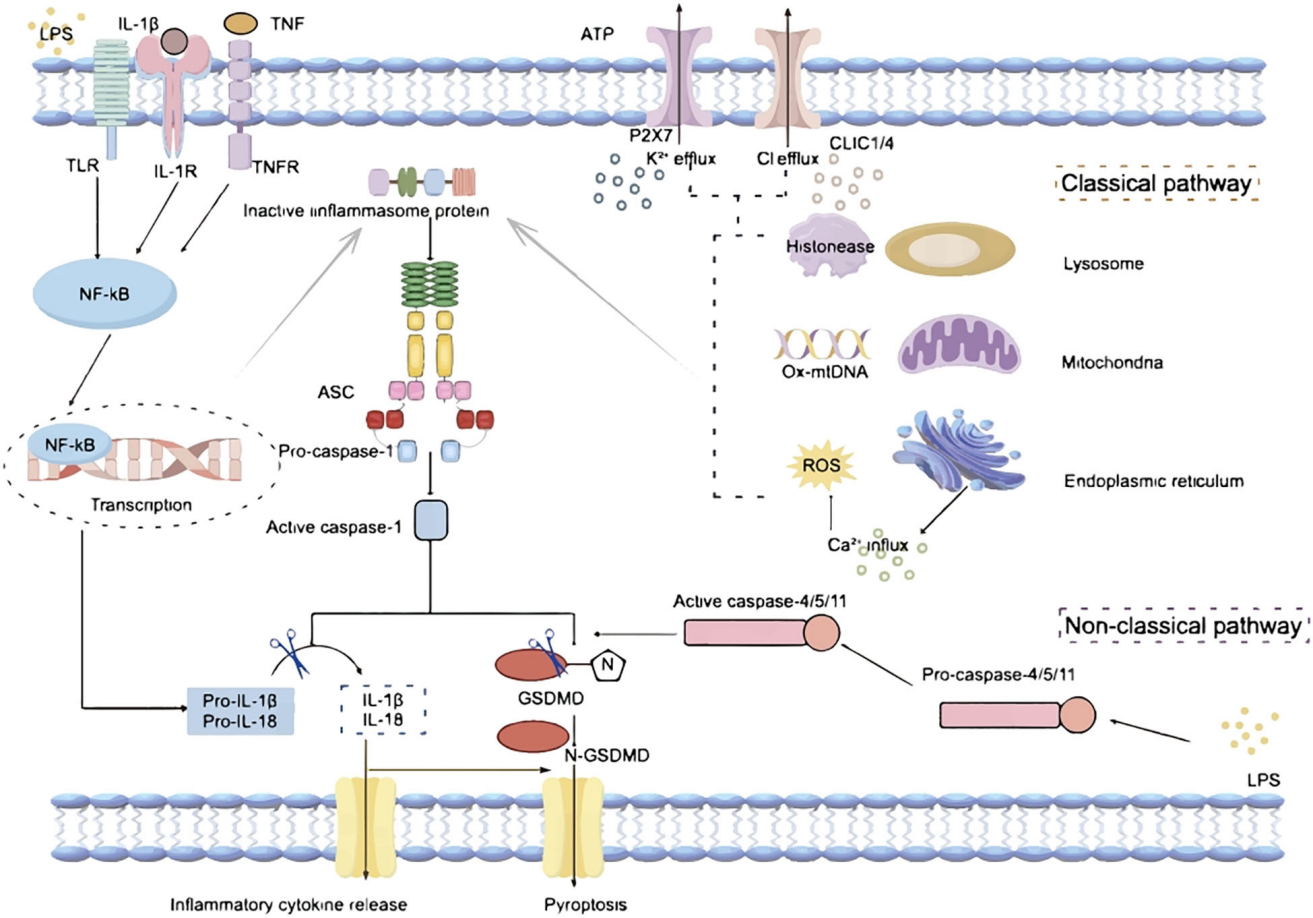
Mechanisms of inflammasome activation. LPS, Lipopolysaccharide; TLR, Toll-like receptor; IL-1β, interleukin-1β; IL-1R, interleukin-1 receptor; IL-18, interleukin-18; TNF, tumor necrosis factor; TNFR, tumor necrosis factor receptor; ATP, Adenosine Triphosphate; P2X7, Purinergic receptor P2X, ligand gated ion channel 7; CLIC1/4, Chloride intracellular channel protein 1/4; NF-kB, nuclear factor-kB; ASC, apoptosis-associated speck-containing protein; Ox-mtDNA, Oxidized mitochondrial DNA; ROS, reactive oxygen species; GSDMD, Gasdermin D.

The inflammasomes serve as pivotal signaling hubs essential for immune regulation and inflammatory responses. Inflammasomes are multi-protein complexes assembled with a receptor protein, such as a nucleotide-binding and oligomerization domain (NOD)-like receptor (NLR) or an absent in melanoma 2 (AIM2)-like receptor protein; and an effector enzyme, typically pro-caspase-1. In classical inflammasomes, the interaction between sensing proteins and effector enzymes is facilitated by the apoptosis-associated speck-containing protein (ASC) (1). Non-canonical pathways are inflammatory responses directly mediated by caspase-4/5 or caspase-11, whose activation does not depend on classical inflammasome receptors but is triggered by the direct recognition of LPS in the cytoplasm (2–4). (**Figure 1**). The receptor in an inflammasome plays a pivotal role in recognizing various pathogen-associated molecular patterns (PAMPs) or danger-associated molecular patterns (DAMPs); and ASC act as adaptor proteins, facilitating inflammasome assembly by binding with the caspase activation and recruitment domain (CARD) of receptors (1, 5). The recruitment of effector, typically pro-caspase-1, leads to its dimerization and autoproteolysis, forming active caspase-1. This cascade induces the cleavage and maturation of interleukin-1β (IL-1β) and IL-18, or the cleavage of gasdermin D (Gsdmd) (6), ultimately triggering the secretion of inflammatory cytokines and a pro-inflammatory form of programmed cell death known as pyroptosis (**Figure 1**).

The activation of an inflammasome requires two steps: priming (Signal 1) and complex assembly (Signal 2). The priming process (Signal 1) is usually triggered by Toll-like receptor (TLR) 4 or tumor necrosis factor (TNF) signaling induced transcription of receptors, pro-IL-1β, and pro-IL-18 via nuclear factor-κB (NF-κB)-dependent pathways (**Figure 1**). The complex assembly (Signal 2) is induced by PAMPs and DAMPs; and numerous molecular or cellular events, including mitochondrial dysfunction related oxidized mitochondria DNA (ox-mtDNA) release and reactive oxygen species (ROS) generation, ion flux (K+/Cl− efflux, and Ca2+ influx), and lysosomal damage, are involved in the inflammasome assembly (**Figure 1**). Among these, K+/Cl- efflux is considered to be the main activation signal of the inflammasomes. The purinergic P2X7 receptor (P2X7R), a cell membrane protein whose activation has been related to a variety of cellular processes, is a nonspecific cation channel that promotes K+ efflux, which in turn mediates the activation of the inflammasome (7–9). In addition, extracellular ATP stimulation of P2X7R triggers K+ efflux, which further induces gradual recruitment of pannexin-1 membrane pores, a process that results in the release of ATP from the intracellular to the extracellular space, thereby amplifying the activation signal (10). The ability of chloride intracellular channel 1 (CLIC1) and 4 (CLIC4) to control Cl- efflux, and the fact that Cl- efflux mainly acts downstream of ROS signaling, further suggests the critical role of CLICs in inflammasome activation (11). While Cl- efflux alone may not be sufficient to fully activate functional inflammasomes, the assistance of K+ efflux is required for subsequent caspase-1 cleavage and IL-1β release (9).Thus, K+/Cl- efflux acts together to synergistically promote inflammasome activation (**Figure 1**).

Recent findings highlight crucial roles of the inflammasome pathways in maintaining immune homeostasis, fighting infections, and regulating inflammatory processes in the body (12). Inflammasomes are classified into NLR and AIM2 inflammasomes based on their sensing proteins. NLR inflammasomes consist of two major families: NLRP (containing the PYD structural domain) and NLRC (containing the CARD structural domain), distinguished by their structural characteristics (13). Key members of the NLRP family include NLRP1, NLRP3, NLRP6, and NLRP12, while the NLRC family includes members such as NLRC4 and NLRC5. The sensing protein of the AIM2 inflammasome is AIM2, characterized by its structure containing the PYD and HIN-200 domains, enabling recognition of pathogen or self-double-stranded DNA (dsDNA) (12). Upon sensing the intracellular signals, these inflammasomes are activated, triggering a cascade of downstream responses, including release of pro-inflammatory cytokines and induction of pyroptosis (6).

Metabolic Syndrome (MetS) is a group of conditions including increased abdominal adiposity, elevated plasma triglycerides (TG), heightened blood pressure, hyperglycemia, and decreased plasma high-density lipoprotein (HDL) levels (14). Diagnosis of MetS is made in obese patients presenting with two or more of the following major risk factors: increased plasma TG, elevated blood pressure, hyperglycemia, and decreased plasma HDL levels (14). MetS is a global health concern, affecting approximately one-fourth of the world’s population and its prevalence is on the rise (15). This increases the risk of type II diabetes mellitus (T2DM) and cardiovascular disease, posing a significant threat to public health and societal well-being (14).

Oxidative stress and chronic inflammation have significant impacts on glucose and lipid metabolism, playing a crucial role in the development of MetS (16, 17). Recent research has highlighted the regulatory role of inflammasomes in oxidative stress and chronic inflammation (18, 19), implicating a potential role of inflammasomes in the onset and progression of MetS. To address this, we are conducting this review to outline the impact of inflammasomes on various aspects of MetS, including obesity, insulin resistance and glucose and lipid metabolism.

## 2 Association between inflammasomes and metabolic syndrome

**Figure 2:**
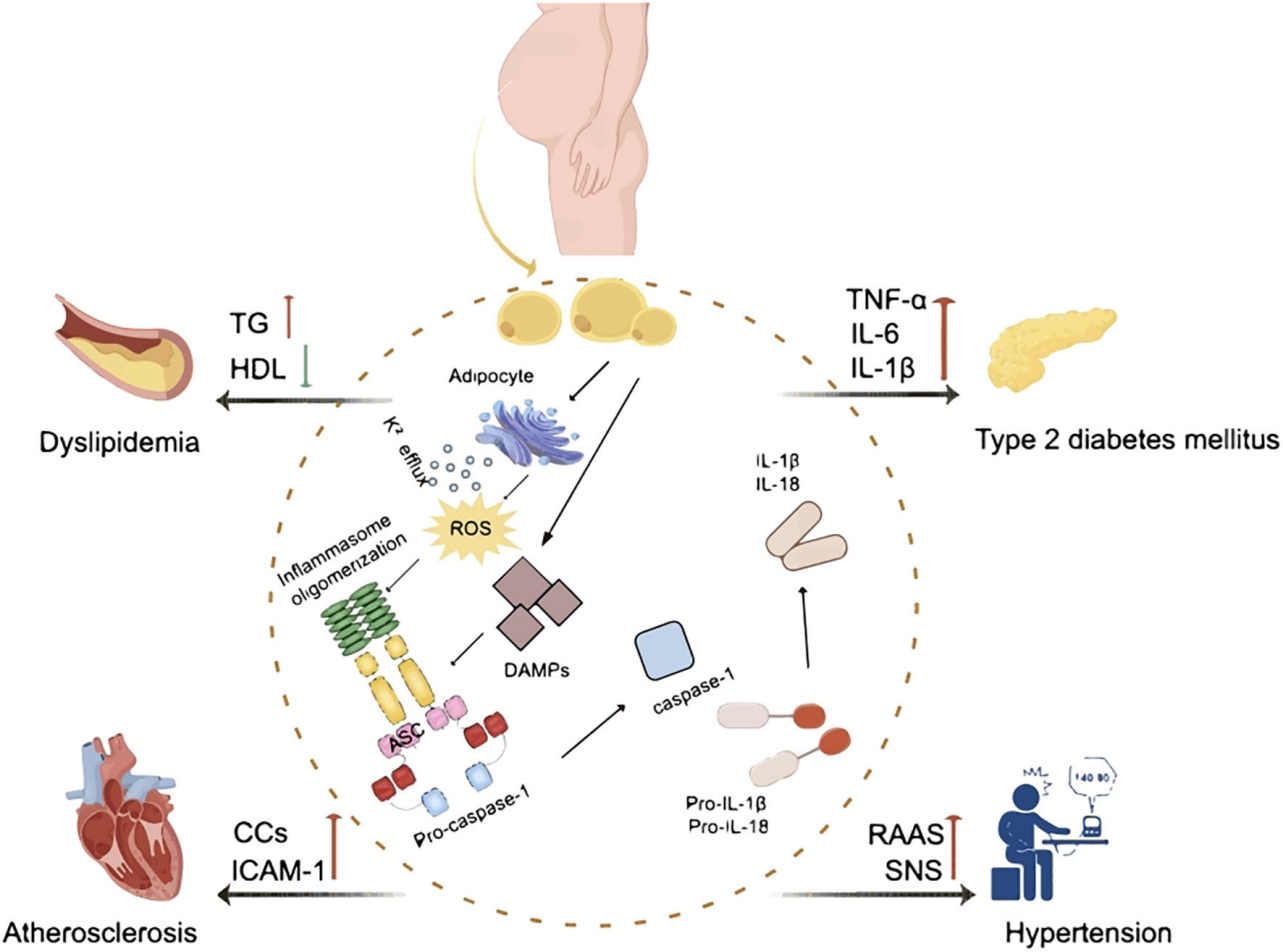
The effect of inflammasomes in metabolic syndrome. ROS, reactive oxygen species; DAMP, damage-associated molecular pattern; ASC, apoptosis-associated speck-containing protein; IL-1β, interleukin-1β; IL-18, interleukin-18; TG, triglycerides; HDL, high-density lipoproteins; TNF-α, tumor necrosis factor α; IL-6, interleukin-6; CCs, cholesterol crystals; ICAM-1, intercellular cell adhesion molecule-1; RAAS, renin-angiotensin-aldosterone system; SNS, sympathetic nervous system.

Obesity induced chronic inflammation is a key component in the pathogenesis of insulin resistance and the MetS. Adipose tissue macrophages (ATM) in obese patients are key players in the regulation of metabolic inflammation. They play a role in the inflammatory and metabolic processes through antigen presentation, activation of adaptive immune cells, and modulation of adipocyte insulin sensitivity (20). It has been shown that the ratio of pro-inflammatory M1 to anti-inflammatory M2 macrophages in obese patients is significantly elevated when treating with lipopolysaccharide (LPS) and interferon-γ (IFN-γ) and is involved in the secretion of pro-inflammatory cytokines such as TNF-α, IL-1β, and IL-6 (21). The increase of TNF-α promoted the activities of IκB kinase (IKK), mitogen activated protein kinase (MAPK), c-Jun N-terminal protein kinases (JNK) and protein kinase C (PKC). These directly target serine residues of insulin receptor substrate (IRS) proteins and impair tyrosine phosphorylation, leading to insulin resistance (22). IL-6 proinflammatory factor-induced JAK-STAT signaling pathway increases the expression of cytokine signaling repressors and related proteins, blocks the interaction of insulin receptor with IRS proteins or alters the kinase activity, and achieves downregulation of insulin receptor function (23–25). In addition, IL-1β activates the downstream MAPK pathway and blocks insulin signaling through serine phosphorylation (26). Increased levels of these pro-inflammatory factors reduce insulin sensitivity, leading to the development of MetS. It’s well known that inflammasomes play a role in infectious diseases and autoimmune diseases. In recent years, a growing body of evidence suggests that inflammasomes also play critical roles in the development and progression of many metabolic disorders including obesity, diabetes, dyslipidemia, hypertension and atherosclerosis (**Figure 2**). This is largely mediated through the increased pro-inflammatory cytokines production and chronic inflammation upon activation. **Table 1** summarized the role and mechanism of different types of inflammasomes in metabolic disorders.

**Table 1:** Inflammasome mechanisms in metabolic syndrome-related diseases.

Diseases	inflammasome	Mechanisms of action	Reference
Type2 diabetes	NLRP3	(1) Causes islet cell dysfunction through IL-1β production, which induces infiltration of pro-inflammatory cells into pancreatic islets and mediates cytokine-induced β-cell apoptosis. (2) Increases the expression of inflammatory mediators such as TNF-α and Mcp1 (Ccl2) by inhibiting the phosphorylation of Akt in primary target tissues	(27–30)
	AIM2	mtDNA is able to activate the AIM2 inflammasome.	(31)
	NLRP6	Induces caspase-11-mediated loss of gut-associated neurons, and microbiota depletion results in the absence of specific intestinal neurons involved in glucose regulation, leading to elevated blood glucose and insulin resistance.	(32, 33),
	NLRC4	Increased secretion of galectin-3 by myeloid cells interferes with insulin signaling, mediated by gasdermin D, leading to insulin resistance.	(34)
Dyslipidemia	NLRP3	Its activation is associated with a reduction in catecholamine-induced TG degradation and the upregulation of genes such as GDF3 and MAOA, which affect TG synthesis and degradation by promoting IL-1β secretion.	(30, 31, 35)
Hypertension	NLRP3	(1) Induction of angiotensin II-induced phenotypic shifts, proliferation, vascular remodeling, and hypertension in vascular smooth muscle cells. (2) The NLRP3/IL-1β and TLR4/NF-κB pathways have important roles in hypertension.	(36–38)
	NLRC4	The nucleotide-derived metabolites adenine and N4-acetylcytidine trigger activation of the NLRC4 inflammasome, leading to IL-1β production, platelet and neutrophil activation, and elevated blood pressure.	(39)
Atherosclerosis	NLRP3	First, oxLDL binds to the CD36-TLR4-TLR6 signaling complex on the macrophage surface and can activate NLRP3 inflammasomes. Second, oxLDL, after being translocated to lysosomes, can activate the NLRP3 inflammasome by inducing cholesterol crystallization in macrophages and consequent lysosomal damage. This in turn activates Caspase-1 and IL-1β and IL-18, triggering cellular pyrolysis.	(40–42)
	AIM2	CAM-1 induces AS by binding to LFA-1 and participating in endothelial monocyte adhesion and monocyte recruitment in AS-prone areas. AIM2 triggers an inflammatory response by triggering vascular smooth muscle cell migration and focal death. Under oxidative stress, the release of mtDNA induces endothelial pyroptosis via the AIM2/caspase-1/GSDMD axis.	(43, 44)

NLRP3, Nod-Like Receptor Protein 3; AIM2, Absent in Melanoma 2; NLRP6, Nod-Like Receptor Protein 6; NLRC4, NLR Family CARD Domain Containing 4; IL-1β, interleukin-1β; TNF-α, tumor necrosis factor α; mtDNA, Mitochondrial DNA; TG, triglyceride; GDF3, growth differentiation factor-3; MAOA, monoamine oxidase A; TLR4, Toll-like receptor4; NF-κB, nuclear factor-k-gene binding; oxLDL, oxidized low-density lipoprotein; ICAM-1, intercellular adhesion molecule-1; LFA-1, lymphocyte function associated antigen-1; AS, Atherosclerosis.

### 2.1 Inflammasomes and obesity

According to World Health Organization, more than 50 percent of the world’s population is now overweight or obese with at least 2.8 million deaths each year attributed to these conditions (45). Obesity is associated with chronic metabolic, and inflammatory alterations in multiple organs, including cardiac, adipose, muscular, hypothalamic, pancreatic, and hepatic tissues. Thus, the risk of serious conditions such as T2DM, hyperlipidemia, fatty liver disease and cardiovascular disease is strongly associated with obesity.

Obesity is characterized by a state of overexpansion of white adipose tissue accompanied by chronic, low-grade inflammation (29). Obesity is associated with immune cell infiltration of adipose tissue and increased production of pro-inflammatory cytokines, leading to adverse effects on normal adipocyte function (46). Endoplasmic reticulum (ER) stress, a process activated in obesity (47, 48), triggers the activation of NLRP3 inflammasome through the generation of ROS and potassium efflux (49). CCAAT/enhancer-binding protein (C/EBP) homologous protein (CHOP), acting as a transcriptional regulator of ER calcium release, amplifies the activation of NLRP3 inflammasome (50). In the context of chronic caloric surplus-induced obesity, elevated levels of DAMPs, including glucose, fatty acids, and reactive lipids, also provoke NLRP3 inflammasome activation (51, 52). Activation of inflammasomes results in infiltration of macrophages and T-cells into adipose tissue, triggering the secretion of IL-1β and IL-18. These cytokines act as master regulators of the inflammatory response, initiating a cascade of other inflammatory mediators that ultimately lead to adipocyte hypertrophy and adipose tissue dysfunction (53–55). It has been shown that the expression levels of caspase-1, IL-1β and IL-18 and caspas-1 activity are significantly elevated in adipose tissue in obese animal models including diet-induced obesity (DIO), *ob/ob* and *db/db* mice (56).

Murphy at al. demonstrated that mice lacking NLRP1 develop spontaneous obesity and MetS, and activation of NLPR1 prevents obesity possibly through regulating IL-18 production (57). This is contradicted to the concept that deletion of major components of inflammasomes prevents obesity and insulin resistance. This could be due to the favorable role of NLRP1 on IL-18 production, as IL-18 deletion has been found to link to obesity and insulin resistance (58). Future studies should be focus on illustrating how NLRP1 balance the production of pro-inflammatory IL-1 β and anti-obesity IL-18.

Our study showed that AIM2 deletion induces spontaneous obesity and insulin resistance in mice. And this is mediated though an inflammasome independent function of AIM2 (59). The NLRP6 inflammasome is implicated in regulating glucose and lipid metabolism through its interactions with the gut-liver axis. It has been demonstrated that activation of NLRP6 promotes IL-18 secretion, which helps maintain intestinal barrier integrity and prevents excessive systemic inflammation that contributes to insulin resistance. In contrast, NLRP6 deficiency exacerbates inflammation in metabolic tissues, promoting the development of obesity, insulin resistance and fatty liver disease (27).

Free fatty acids have distinct effects on NLRP3 inflammasome. The saturated fatty acid, palmitate, diminishes mitochondrial adenosine monophosphate-activated protein kinase (AMPK) activity, impairs autophagy and promotes ROS accumulation, thereby inducing NLRP3 inflammasome activation (28). Moreover, saturated fatty acids directly stimulate TLR2, inducing the expression of NLRP3 via nicotinamide adenine dinucleotide phosphate (NADPH) oxidase-dependent pathway and nuclear factor kappa B (NF-κB) activation (30). This is similar to the effect observed with TLR4 activation (35). Conversely, unsaturated fatty acids inhibit the activation of NLRP3 inflammasome by preserving mitochondrial AMPK activity, thereby hindering IL-1β processing (31, 60). The ketone body, β-hydroxybutyrate (BHB) impedes NLRP3 inflammasome activation by restraining potassium efflux and reducing ASC oligomerization and speck formation (32).

Taken together, obesity-induced inflammasome activation contributes the inflammatory response in the body and induces the development of other metabolic disorders. Therefore, inflammasomes are a key component in the formation of a chronic low-grade inflammatory environment in obesity.

### 2.2 Inflammasomes and diabetes

Hyperglycemia is a common feature for both type 1 diabetes mellitus (T1DM) and type 2 diabetes mellitus (T2DM). T1DM is an autoimmune disease characterized by an attack on pancreatic β cells by the immune system, resulting in decreased or complete halt in insulin production. T2DM is typically characterized by chronic low-grade inflammation and insulin resistance. It’s been reported that hyperglycemia correlates with inflammasome dysregulation (33, 34). Inflammasomes play pivotal roles in both pancreatic β-cell damage and insulin resistance (**Table 1**).

Inhibition of caspase-1 using genetic or chemical approaches reduces body weight gain and improves insulin sensitivity in high-fat diet (HFD)-induced obesity mouse model (56). Similarly, deficiency in other key components of inflammasome such as ASC and NLRP3 also protects mice from HFD induced weight gain and insulin resistance (53). In addition, calorie restriction and exercise-mediated weight loss and improved insulin sensitivity in obese individuals with T2DM is associated with reduced expression of NLRP3 and inflammation markers (61).

It has been shown that NLRP3 inflammasome activation causes pancreatic islet cell dysfunction primarily through IL-1β production. This induces the infiltration of pro-inflammatory cells to pancreatic islets. Cytokines secreted from the immune cells can induce apoptosis of β-cells (62, 63). Knockdown of NLRP3 inhibits T cell activation, Th1 cell differentiation, and the infiltration of pathogenic T-cell to pancreatic islets by decreasing the expression of chemokines in islet T cells and non-hematopoietic cells (64).

It has been shown that there is an increase in expression levels of AIM2 and IL-1β in monocytes in patients with T2DM compared to healthy controls, along with elevated serum levels of cellular mtDNA. This suggests that increased levels of circulating mtDNA and AIM2 inflammasome activation may contribute to the inflammatory process in patients with T2DM (65). Studies have shown that mtDNA release during mitochondrial stress caused by infection or potential dietary lipid overload may induce the assembly of AIM2 inflammasome (66). These results suggest that increased AIM2 expression, elevated circulating mtDNA levels, and AIM2 inflammasome activation may be involved in the inflammatory process in patients with T2DM. NLRP6 inflammasome activation induces caspase-11-mediated loss of gut-associated neurons. Microbiota depletion results in the absence of specific intestinal neurons involved in glucose regulation, leading to elevated blood glucose and insulin resistance (67, 68). Pearson and colleagues showed that NLRP6 deletion protects mice from T1DM in mice through promoting regulatory B cell population (69). Interestingly, the authors also found that gut microbiota is critical for the induction of regulatory B cells in NLRP6-deficiency mice, and NLRP6-deficiency is required to maintain the function of regulatory B cells (69). The activation of NLRC4 inflammasome increases secretion of galectin-3 by myeloid cells through gasdermin D, thereby causing insulin resistance (70). It has been shown that single-nucleotide polymorphism of NLRP1 is associated with autoimmune diseases, including T1DM (71). Costa et al. showed that NLRP1 expression suppresses Th17 differentiation and inhibits IL-17 production in T1DM in mouse and human (33). Soares and colleagues demonstrated that NLRP1 gain-of-function variants protect against diabetic kidney disease in humans, highlighting an emerging protective role of NLRP1 against metabolic stress (72). It needs more studies to clarify whether this is also mediated through the production of IL-18.

Taken together, these findings suggest that inflammasomes, including NLRP3, AIM2, NLRP1, NLRP6 and NLRC4 inflammasomes are involved in the regulation of insulin resistance, pancreatic β-cell health and diabetes. Therefore, targeting these inflammasomes may provide new therapeutic strategies for treating diabetes. Future studies should also focus on investigating whether other inflammasomes are also involved in the onset or progression of T1DM or T2DM; and whether inhibition of key component of inflammasomes improves insulin resistance and diabetes.

### 2.3 Inflammasomes and dyslipidemia

Lipids are a general term for neutral fats (mainly triglycerides) and lipids (phospholipids, glycolipids, sterols, steroids) in plasma, and they play important roles in human metabolism. Among them, triglycerides are mainly responsible for the energy metabolism of the body. Epidemiologic findings show that obesity leads to dyslipidemia by increasing plasma levels of TG and decreasing levels of high-density lipoproteins (HDL) (73) (**Table 1**).

Inflammasomes play a pivotal role in dyslipidemia, affecting both TG and HDL levels. The activation of NLRP3 inflammasome is associated with a decrease in catecholamine-induced TG degradation, possibly through the upregulation of genes such as growth differentiation factor-3 (GDF3) and monoamine oxidase A (MAOA) (36). Inflammasomes also influence TG homeostasis by promoting IL-1β production (37). Notably, IL-1β has been implicated in inducing hypertriglyceridemia by inhibiting the activity of lipoprotein lipase (LPL) and suppressing lipogenesis (74). Furthermore, caspase-1, a key component of inflammasomes, has an impact on lipid metabolism. Kotas et al. (75) demonstrated that caspase-1-deficient mice exhibit accelerated TG clearance without altering lipid production or uptake during a fat tolerance test using olive oil. This leads to a decrease in steady-state circulating TG and fatty acid levels (75).

Cholesterol transporter proteins, specifically ATP-binding cassette transporters A1 and G1 (ABCA1/G1), are responsible for cholesterol efflux to HDL. The enrichment of cholesterol in the plasma membrane and enhanced TLR4 signaling in Abca1/Abcg1-deficient macrophages suggest the initiation of inflammasome activation (38). On the other hand, HDL inhibits the activation of inflammasomes by reducing the expression of pro-IL-1β and NLRP3, which in turn attenuates the activation of caspase-1 (76). However, it is still unclear whether inflammasome activation leads to reduced HDL levels due to the anti-inflammatory properties of HDL.

### 2.4 Inflammasomes and hypertension

Hypertension has been increasingly linked to multiple inflammasomes, including NLRP3, AIM2, and NLRC4, contributing to vascular dysfunction and blood pressure regulation. Chronic vascular inflammation promotes endothelial dysfunction, arterial stiffness, and increased sympathetic nervous system activity, all of which contribute to hypertension. IL-1β and IL-18, the inflammasome-induced cytokines play critical roles in hypertension (77, 78) and inhibition of IL-1β has shown potential in reducing blood pressure and preventing the development of hypertension (79). Polymorphisms in the NLRP3 gene have been identified as potential factors contributing to increased blood pressure levels. It has been shown that individuals with NLRP3 polymorphisms tend to have higher blood pressure levels compared to those without such genetic variants after the age of 50 (39). A 42-base variable number of tandem repeats (VNTR) polymorphism in NLRP3 has been linked to an increased susceptibility to essential hypertension (80). Studies have shown that genetic knockout of NLRP3 or ASC prevents blood pressure elevation in the two-kidney, one-clip (2K1C) model of hypertension in mice (81). This further supports the pivotal role of NLRP3 inflammasome in the development of hypertension. NLRP3 inflammasome activation contributes to angiotensin II-induced vascular smooth muscle cell phenotypic transformation, proliferation, vascular remodeling, and hypertension (82). Inhibition of the AMPK-NLRP3 inflammasome-high mobility group box (HMGB)-1 axis provides protection to endothelial cells (83). The development of hypertension often involves complex processes, including renal injury, vascular remodeling, and modulation of the paraventricular nucleus (PVN) of the hypothalamus. Inflammasomes play a crucial role in these processes, influencing renal injury, vascular remodeling, and hypothalamic PVN regulation. Chronic inhibition of NF-κB activity in the PVN of salt-sensitive hypertensive rats delays the development of hypertension through reducing NLRP3 and IL-1β expression, as well as attenuating the activity of IKKβ and NAD(P)H oxidase (40). NLRP3 activation in the hypothalamic PVN contributes to sympathetic nervous system overactivity, increasing blood pressure (84). And blocking of NLRP3 inflammasome in the brain attenuates salt-induced prehypertension, potentially through modulating inflammation-induced cascade responses and restoring neurotransmitter homeostasis (84). Recent research using a rat model of hypertension demonstrated that NF-κB blockade reduces blood pressure elevation and protects against hypertension-induced renal injury. This study also highlights the importance of inflammasome activation in hypertension, with concurrent involvement of the NLRP3/IL-1β and TLR4/NF-κB pathways in promoting kidney injury and inflammation (41).

Other inflammasomes, such as the NLRC4 inflammasome, have been tentatively linked to the regulation of blood pressure in mice. Nucleotide-derived metabolites, adenine and N4-acetylcytidine, detectable in blood, trigger the activation of NLRC4 inflammasome, leading to IL-1β production, platelet and neutrophil activation, and blood pressure elevation in mice (42). A single nucleotide polymorphism within NLRP6 is associated with decreased susceptibility to hypertension in males (43). Although a direct link between AIM2 inflammasome and hypertension has not been established, it has been shown that AIM2 is related to endothelial dysfunction, a significant factor in the development and progression of hypertension (85). Interestingly, chrysanthemum indicum extract, a traditional herbal medicine using for hypertension, inhibits NLRP3 and AIM2 inflammasome activation via regulating ASC phosphorylation in mice (86). These findings highlight the multifaceted role of inflammasomes in hypertension pathogenesis and suggest that targeting inflammasome pathways, beyond just NLRP3, may offer novel therapeutic strategies for hypertension management. Compared to other inflammasomes, the role of NLRP3 inflammasome in hypertension has been extensively studied. Future study should focus on how activation of other inflammasomes is involved in the various network systems in hypertension.

#### 2.4.1 Obesity-hypertension comorbidity mechanisms

Sixty to seventy percent of primary hypertension can be attributed to obesity. It has been shown that every 4.5 kg increase in body weight increases systolic blood pressure by 4 mmHg (87). White fat expansion, local tissue hypoxia, ER stress response, and infiltration of immune cells (especially macrophages) in adipose tissue in obesity can activate inflammasome, leading to chronic inflammation. The inflammatory response triggered by obesity can lead to adipose tissue lesions, activation of the renin-angiotensin-aldosterone system (RAAS), sympathetic nervous system (SNS) hyperactivity, insulin resistance, vascular dysfunction and renal pathology, thereby elevating blood pressure (88–90). Increased leptin secretion from adipose tissue in obesity induces the secretion of angiotensin II and activates the SNS, therefore causing increased blood pressure. Increased JNK levels and insulin resistance in obesity also cause hyperactivation of the SNS (91, 92). In addition, adipose tissue secreted pro-inflammatory cytokines, including IL-6, IL-1β and TNF-α induce changes in blood lipid levels, therefore, causing changes in the cardiovascular system and leading to hypertension (93).

### 2.5 Inflammasomes and atherosclerosis

AS is a chronic inflammatory disease caused by lipid accumulation and inflammation in large arteries and is a leading cause of death worldwide (94). Previous studies have shown that obesity is an independent risk factor for AS, and it contributes to the formation and progression of AS alone or in association with other factors (95) (**Table 1**).

Inflammation-induced endothelial damage, accumulation of oxidized low-density lipoprotein and cholesterol crystals in the vessel wall, and recruitment and proliferation of smooth muscle cells are the major pathogenic causes of AS (96, 97). Studies (98) have shown that the presence of large amounts of cholesterol crystals and oxidized low-density lipoprotein (oxLDL) in AS plaques activate the NLRP3 inflammasome, which in turn induces the maturation of inflammatory cytokines and triggers cellular pyroptosis. Duewell et al. (99) found that cholesterol crystals (CCs) in early AS lesions are proportional to the presence of inflammatory cells, again demonstrating the importance of the NLRP3 inflammasome in the development and progression of AS. Grebe and colleagues (100) showed that in a mouse model of AS, oxLDL first binds to the CD36-TLR4-TLR6 signaling complex on the surface of macrophages to initiate the NLRP3 inflammasome. Other studies have shown that oxLDL, after being transported to lysosomes, activates the NLRP3 inflammasome by inducing cholesterol crystallization and concomitant macrophage lysosomal damage (101).

Except for the NLRP3 inflammasome, the AIM2 inflammasome was also found to play a role in AS. Pan et al. (102) showed that AIM2 increases the levels of intercellular adhesion molecule (ICAM)-1 in apolipoprotein (Apo) E knockout mice. This is involved in endothelial monocyte adhesion and monocyte recruitment in AS-prone areas by binding to lymphocyte function-associated antigen (LFA)-1, therefore, inducing AS (102). It has been shown that AIM2 expression levels increased with elevated oxidative DNA damage and DNA replication stress in atherosclerosis-prone *JAK2^VF^* mice. Under oxidative stress, the release of mtDNA induces endothelial pyroptosis via the AIM2/caspase-1/GSDMD axis (44). Deletion of AIM2 or Gsdmd reduces atherosclerosis, suggesting a role of AIM2-mediated pyroptosis in AS (103). In addition, AIM2 inflammasome triggers vascular smooth muscle cell migration and death and promotes inflammatory response in AS lesions by facilitating the release of proinflammatory cytokines (104). Borborema et al. reported that the expression levels of NLRP1 and NLRC4 are significantly higher in the peripheral blood in patients with AS compared to the healthy controls (105) indicating a potential role of these inflammasomes in AS.

The Canakinumab Anti-inflammatory Thrombosis Outcomes Study (CANTOS) trial (106, 107) is an important milestone in the field of cardiovascular disease in recent years. It is the first definitive demonstration that targeting the IL-1β pathway significantly reduces the risk of relapse in patients with atherosclerotic cardiovascular disease. The trial showed that patients treated with canakinumab had a significantly reduced risk of nonfatal myocardial infarction, nonfatal stroke, or cardiovascular death, especially in the high-dose group. However, despite canakinumab’s excellent performance in reducing cardiovascular events, its potential side effects should not be overlooked. In this study, the incidence of fatal infections increased in the canakinumab group compared to the placebo group, suggesting that the risk of immunosuppression may be associated with inflammation-targeted therapy. In addition, although canakinumab did not significantly reduce LDL-C levels, its significant reduction in C-reactive protein (CRP) levels may indirectly reflect improved inflammatory status. Therefore, while promoting inflammation-targeted therapies as a management strategy for atherosclerotic disease, it is important to weigh their potential immunosuppressive risks and further explore safer and more effective treatments.

In summary, NLRP3 and AIM2 inflammasomes play important roles in the development of AS. However, no direct evidence for the use of AIM2 inflammasome in the prevention and treatment of AS, and whether their inhibition can be an effective target in the prevention and treatment of AS still requires in-depth research and demonstration.

## 3 Influence of metabolic syndrome on inflammasomes

MetS and inflammasomes are not just a one-way relationship in which one activates the other; numerous studies have shown that the two can act as upstream and downstream of each other to promote the activation of inflammatory factors, resulting in the generation of a chronic, low-grade inflammatory environment in the body. A distinctive characteristic of MetS is the emergence of an inflammatory environment, referred to as “metaflammation”. This leads to the assembly and activation of inflammasomes, triggering the early innate immune response. The induced expression levels of pro-IL-1β and NLRP3 is the first step of NLRP3 inflammasome activation.

HMGB1 activates p38 MAPK and NF-κB via the receptor for advanced glycation end products (RAGE), promoting the synthesis of pro-IL-1β and pro-IL-18 in THP-1 macrophages (108). In T2DM, the aggregation of islet amyloid polypeptide (IAPP) in the pancreas triggers the activation of NLRP3 inflammasome (109). Thioredoxin-interacting protein (TXNIP), a protein associated with insulin resistance, also plays a role in NLRP3 inflammasome activation (110). Under normal conditions, TXNIP binds to and inhibits thioredoxin (TRX), but in the high-glycemic environment of T2DM, TXNIP dissociates from TRX and interacts with and activates the NLRP3 inflammasome in different cell types (111).

Uncontrolled hyperglycemia triggers the formation of advanced glycation end products (AGEs) through nonenzymatic glycosylation reactions between reducing sugars and proteins, lipids, or nucleic acids (112, 113). Under conditions of chronic hyperglycemia, AGEs accumulate in circulation and tissues, forming irreversible cross-links among various intracellular and extracellular molecules. The activation of the RAGE by AGEs triggers downstream signaling pathways, generating ROS and inducing oxidative stress (114, 115). Moreover, intracellular glycosylation of mitochondrial respiratory chain proteins by AGEs exacerbates ROS production, creating a vicious cycle that promotes endogenous AGE production. RAGE mediates the activation of AIM2 inflammasome by regulating double-stranded RNA-dependent protein kinase phosphorylation in macrophages (116).

Activation of obesity-associated chronic inflammatory pathways can damage vascular endothelial cells, leading to vascular dysfunction and further causing hypertension. The development of hypertension can also lead to exacerbation of vascular dysfunction, creating a vicious cycle. The entire process begins with the activation of inflammasomes by obesity, which triggers the secretion of pro-inflammatory factors. This contributes to the development of Mets such as insulin resistance, hypertension, dyslipidemia, and AS. Following the onset of MetS, the activation of inflammasome further amplifies the inflammatory milieu, creating self-reinforcing positive feedback loops. These loops are characterized by a continuous increase in intracellular inflammatory factors and a persistent exacerbation of inflammatory responses, perpetuating the cycle of inflammation and metabolic dysfunction. These findings highlight the complex interplay of metabolism and inflammasomes in the pathogenesis of MetS.

Inflammasomes interact with various metabolic regulatory pathways, including the mechanistic target of rapamycin (mTOR) and peroxisome proliferator-activated receptor gamma (PPARγ), highlighting the complexity of their roles in metabolic disorders. The mTOR pathway, a central regulator of cell growth and metabolism, modulates inflammasome activation in response to metabolic stress. mTORC1 has been shown to regulate NLRP3 inflammasome activation by controlling autophagy and mitochondrial homeostasis. Under conditions of nutrient excess, mTORC1 suppresses autophagy, leading to the accumulation of damaged mitochondria and increased ROS, which serve as potent triggers for inflammasome activation (117). Conversely, inhibition of mTORC1 by rapamycin promotes autophagic clearance of inflammasome activators, thereby reducing inflammation (118).

PPARγ, a nuclear receptor involved in lipid metabolism and insulin sensitivity, also regulates inflammasome activity. As a key transcriptional regulator of adipogenesis and glucose homeostasis, PPARγ exerts anti-inflammatory effects by inhibiting NF-κB signaling. This may in turn suppress the expression of NLRP3 and IL-1β. Yang and colleagues showed that PPARγ agonist attenuates caspase-1 and IL-1β maturation during NLRP3 inflammasome activation. The authors also demonstrated that PPARγ suppresses NLRP3 inflammasome activation through direct interaction with NLRP3 (119). Meng et al. reported that PPARγ activation inhibits NLRP3 inflammasome in neurons in a NF-κB independent manner (120). Furthermore, PPARγ plays a role in mitochondrial function and oxidative metabolism, suppresses the accumulation of ROS, a known activator of NLRP3 (121). On the other hand, PPARγ is required for AIM2 expression during M. tuberculosis infection in human macrophages. Although AIM2 is not important for IL-1β release, PPARγ is essential for IL-1β release in human macrophages (122). Kempster et al. reported that PPARγ agonist, rosiglitazone, treatment increases NLRP6 expression in human intestinal epithelial cells (123).

These interactions underscore a complex relationship between metabolism and inflammasomes. Future research should explore dual-target strategies that modulate both inflammasomes and metabolic pathways to develop more effective therapeutic approaches for metabolic syndrome and related diseases.

## 4 Targeting inflammasomes for the metabolic syndrome

The pursuit of specific pharmacological interventions is crucial in reducing the social and economic burdens associated with MetS. Inflammation and oxidative stress signals, serve as attractive targets for anti-inflammatory therapies. However, targeting on these pathways may not always yield significant therapeutic effects due to the complexity of these pathways (37, 124). This highlights the potential benefits of focusing therapeutic efforts on the inflammasome pathways, paving the way for promising future research. Both established drugs currently in clinical use and newly developed compounds yet to be applied clinically show potential in treating MetS by modulating inflammasome pathways (**Table 2**).

**Table 2:** Targeting inflammasomes for the metabolic syndrome.

Treatment	Mechanisms of action	Clinical development stage	Reference
Metformin	Activation of the AMPK pathway inhibits caspase-1 and IL-1β, thereby increasing insulin sensitivity.	Approved	(88)
Resveratrol	Inhibition of NLRP3 inflammasome activation via Sirt1 and Sirt6.	Dietary supplement	(88, 89)
Verapamil	Intervenes in the pathogenesis of islet injury by inhibiting TLR4, TXNIP and NLRP3 inflammasome.	Approved	(90)
γT3	Inhibits NLRP3 inflammasome by inducing A20/TNF-α-interacting protein 3 and activating the AMPK/autophagy axis.	Phase II trials	(91)
Flavopiridol	Inhibits palmitate-induced activation of NLRP3 inflammasome and ameliorates obesity-related inflammation by inducing macrophage autophagy.	Most trials halted	(92)
Chamomile lactone	An NLRP3 inflammasome inhibitor.	Preclinical	(93)
MCC950	An inflammasome inhibitor that interacts with the Walker B structure in NLRP3 NACHT, which in turn blocks ATP hydrolysis and inhibits NLRP3 inflammasome assembly and activation.	Preclinical	(94)
CY-09	Interacts directly with the walker A structural domain of NLRP3 and inhibits the binding of ATP to NLRP3.	Preclinical	(95)
Tretinoin	Can reduce NLRP3 inflammasome and pro-inflammatory factors by reducing them.	Approved	(96)

NLRP3, Nod-Like Receptor Protein 3; AMPK, AMP-activated protein kinase; IL-1β, interleukin-1β, Sirt1, silent mating type information regulation 2 homolog 1; Sirt6, silent mating type information regulation 2 homolog 6; TLR4, Toll-like receptor 4; TXNIP, interacting protein; γT3, γ-tocotrienol; NACHT, oligomerized structural domain.

Therapeutic approaches targeting on inflammasomes for diabetes have attracted significant attention. Lee et al. (125) demonstrated that metformin inhibits caspase-1 and IL-1β through activation of AMPK pathway, thereby enhancing insulin sensitivity. Metformin has also been shown to improve insulin resistance by reducing expression levels of ASC and caspase-1 in a rat model of T2DM (126). Resveratrol may suppress the NLRP3 inflammasome through the activation of Sirt1 and Sirt6, thereby improving insulin resistance (126, 127). Verapamil, a calcium channel blocker, intervenes the pathogenesis of pancreatic islet injury primarily through inhibiting TLR4, TXNIP, and NLRP3 inflammasome, demonstrating the antidiabetic potential (128). γ-Tocotrienol (γT3) inhibits NLRP3 inflammasome through the induction of A20/TNF-α-interacting protein 3 and the activation of AMPK/autophagy axis, thereby slowing the progression of T2DM (129).

In the context of obesity, several therapeutic approaches targeting inflammasomes have been explored. Flavopiridol suppresses the activation of NLRP3 inflammasome induced by palmitate and improves obesity-related inflammation and insulin resistance through inducing macrophage autophagy (130). Chamomile lactone, an NLRP3 inflammasome inhibitor, shows potential as a therapeutic agent for reducing obesity-induced insulin resistance (131). MCC950, a selective NLRP3inflammasome inhibitor, interacts with walker B structure in the nucleotide-binding and oligomerization domain (NACHT) of NLRP3, which in turn hinders ATP hydrolysis and inhibits NLRP3 inflammasome assembly and activation, thus enhancing insulin sensitivity in mice (132). CY-09, another NLRP3 inflammasome inhibitor, interacts directly with the walker A structural domain of NLRP3, inhibiting ATP binding to NLRP3. A recent study demonstrated that combining gastric thoracoplasty with CY-09 reduces body weight, improves insulin resistance, and reduces hepatic steatosis in mice fed with HFD (133). Tretinoin has been shown to improve hyperglycemia, insulin deficiency, impaired glucose tolerance, and insulin resistance in gestational diabetes by decreasing NLRP3 inflammasome and pro-inflammatory factors (134). Compound C significantly reduces insulin resistance in HFD-induced obese mice by downregulating components of NLRP3 inflammasome and pro-inflammatory markers (135). Niacin, a vitamin used in the treatment of dyslipidemia, attenuates experimental nonalcoholic steatohepatitis by inhibiting the NLRP3 inflammasome/focal death pathway (136).

In the mice with hypertension, MCC950 has been observed to reduce the expression of NLRP3 inflammasome subunits and markers of inflammation, and kidney injury, suggesting that MCC950’s ability to lower blood pressure in salt-induced hypertension is mediated through the suppression of inflammasome activity (137, 138).

To date, most of the pharmacological inhibition of inflammasome activity studies are focused on the NLRP3 inflammasome. Future studies should be carried out to develop novel inhibitors for other inflammasomes such as NLRC4 and AIM2 inflammasomes, and to study how inhibition of these inflammasome pathways affect MetS.

## 5 Conclusion

The activation of inflammasomes following the onset of obesity, hyperglycemia, hypertension, and dyslipidemia forms a pivotal pathway and signaling cascade that promotes chronic inflammation and increased oxidative stress. The immune modulation and inflammatory response controlled by these inflammasomes play a critical role in the complex signaling networks that drive the development of MetS. As MetS progresses, the enhanced oxidative stress and accumulation of AGEs further stimulate the activation of inflammasomes, thereby intensifying chronic inflammation. As a result, interventions targeting on inflammasomes show potential in attenuating the incidence of MetS and slowing down the persistent progression of chronic inflammation associated with MetS. This provides novel tools and therapeutic targets for clinical intervention of metabolic diseases. To date, the role of NLRP3 in MetS including obesity, diabetes, dyslipidemia, hypertension and AS is well studied. research on the role of other inflammasomes in MetS is still emerging. Most of the known chemical inhibitors that demonstrate a role in MetS are targeting on NLRP3 inflammasome. AIM2, as the sensor of dsDNA, is drawing more attention nowadays, as oxidative stress and DNA damage are found to play pivotal role in metabolic disorders. In summary, studies should be carried out to illustrate the role and mechanism of other inflammasomes, including AIM2, NLRP1, NLRP6 and NLRC4 inflammasomes, in metabolic disorders.

### 5.1 Future directions

High throughput or high content screening studies should be conducted to discover selective inhibitors for other inflammasomes and test their roles in MetS. The development of dual-target molecules targeting on both inflammasomes and metabolism are particularly important in the field of MetS. In addition, understanding the cell-specific and tissue-specific functions of inflammasomes is essential for creating targeted treatments for diseases linked to inflammasome dysregulation. While inhibiting inflammasomes with chemical inhibitors or antibodies can be a promising therapeutic strategy, it’s important to note that this approach can lead to unintended immunosuppressive effects, because inflammasomes are an important component of innate immunity. Thus, identifying the side effects of inflammasome inhibitors is an urgent priority. Due to the divergent roles of IL-1 β and IL-18 in immunity and metabolism, studies on how each inflammasome balances the production of these cytokines could also be an attractive direction.
